# Conserved and Divergent Principles of Planar Polarity Revealed by Hair Cell Development and Function

**DOI:** 10.3389/fnins.2021.742391

**Published:** 2021-10-18

**Authors:** Michael R. Deans

**Affiliations:** ^1^Department of Surgery, Division of Otolaryngology, University of Utah School of Medicine, Salt Lake City, UT, United States; ^2^Department of Neurobiology, University of Utah School of Medicine, Salt Lake City, UT, United States

**Keywords:** hair cell, vestibular, auditory, planar cell polarity (PCP), planar polarity

## Abstract

Planar polarity describes the organization and orientation of polarized cells or cellular structures within the plane of an epithelium. The sensory receptor hair cells of the vertebrate inner ear have been recognized as a preeminent vertebrate model system for studying planar polarity and its development. This is principally because planar polarity in the inner ear is structurally and molecularly apparent and therefore easy to visualize. Inner ear planar polarity is also functionally significant because hair cells are mechanosensors stimulated by sound or motion and planar polarity underlies the mechanosensory mechanism, thereby facilitating the auditory and vestibular functions of the ear. Structurally, hair cell planar polarity is evident in the organization of a polarized bundle of actin-based protrusions from the apical surface called stereocilia that is necessary for mechanosensation and when stereociliary bundle is disrupted auditory and vestibular behavioral deficits emerge. Hair cells are distributed between six sensory epithelia within the inner ear that have evolved unique patterns of planar polarity that facilitate auditory or vestibular function. Thus, specialized adaptations of planar polarity have occurred that distinguish auditory and vestibular hair cells and will be described throughout this review. There are also three levels of planar polarity organization that can be visualized within the vertebrate inner ear. These are the intrinsic polarity of individual hair cells, the planar cell polarity or coordinated orientation of cells within the epithelia, and planar bipolarity; an organization unique to a subset of vestibular hair cells in which the stereociliary bundles are oriented in opposite directions but remain aligned along a common polarity axis. The inner ear with its complement of auditory and vestibular sensory epithelia allows these levels, and the inter-relationships between them, to be studied using a single model organism. The purpose of this review is to introduce the functional significance of planar polarity in the auditory and vestibular systems and our contemporary understanding of the developmental mechanisms associated with organizing planar polarity at these three cellular levels.

## Introduction

Planar polarity is the coordinated organization of cellular polarities within the plane of an epithelial layer. It is frequently manifest in the organization of apical structures such as the stereociliary bundles of vertebrate mechanosensory hair cells that are the focus of this review, or the trichome hairs projecting from *Drosophila* wing cells that have been a longstanding model for planar polarity research (Goodrich and Strutt, 2011). Small cohorts of cells or functional cell clusters are also often organized as planar polarized groups. For example, the hair follicles distributed throughout mammalian skin consist of cell clusters coordinately oriented to project a single hair in a uniform direction (Devenport and Fuchs, 2008), and the chiral organization of photoreceptors in the ommatidia of the insect’s compound eye has a stereotyped planar polarity reflecting ommatidia position (Jenny, 2010). These examples share a common two-fold organization in which cells or cellular clusters are intrinsically polarized and that polarity is coordinated with neighboring cells or clusters throughout the epithelium. The mechanisms coordinating neighboring cells are largely conserved between tissues and across species, and are encoded by the Planar Cell Polarity (PCP) genes (Goodrich and Strutt, 2011). These genes function upstream of the cell and tissue-specific effectors that mediate cell-intrinsic polarity. In vertebrates, PCP gene function has also been linked to other developmental processes such as synapse formation and axon guidance (Nagaoka et al., 2014; Thakar et al., 2017), but it is unclear how much this reflects a conserved planar polarity activity or the repurposing of signaling molecules whose primary function in *Drosophila* is restricted to planar polarity. Conversely, vertebrate genes not commonly associated with planar polarity in *Drosophila* have clear roles in planar polarization during vertebrate development (Montcouquiol et al., 2003; Lu et al., 2004). For the purposes of this review, any cellular process or structure that is organized parallel to the plane of an epithelium will be considered planar polarized, and any proteins contributing to their maintenance or development have planar polarity functions.

Planar polarization is essential for the auditory and vestibular functions of the vertebrate inner ear because it underlies the mechanism by which mechanosensory hair cells detect sound or motion. Projecting from the apical surface of hair cells is a bundle of stereocilia arranged in rows of increasing height with the tallest positioned next to a single, laterally displaced kinocilium. Stereocilia are specialized microvilli composed of filamentous actin, whereas the kinocilium is a true tubulin-based cilium anchored at a basal body located beneath the apical cell surface (Schwander et al., 2010). The planar polarity of individual hair cells is manifest in two ways, the orientation of the staircase of stereocilia progressing from rows of shorter to longer length, and the lateral position of the kinocilium. This organization underlies hair cell function because stereocilia in adjacent rows are interconnected by protein crosslinks called tip-links that couple mechanically gated ion channels on the tips of shorter stereocilia with the shafts of their taller neighbors. As a result, mechanical stimuli deflecting the bundle toward the kinocilium generates tension that opens these channels and depolarizes the cell. Deflections in the opposite direction reduces tension, closing channels and moving the hair cell to a more hyperpolarized state. This mechanism underlies mechanotransduction in both auditory and vestibular hair cells, and in each the stereocilia are deflected by overlying extracellular matrices that move in response to sound or motion (Hudspeth, 1989; Gillespie and Muller, 2009). As a result, there is a direct link between the planar polarization of the stereociliary bundle and mechanotransduction, and building a polarized bundle that is properly oriented relative to these extracellular matrices is essential for hearing and balance function.

The planar polarized organization of hair cell stereociliary bundles can be visualized at three levels. At a cellular level, intrinsic planar polarity is readily evident in the organization of stereocilia and kinocilium within the bundle. At a tissue level, planar polarity is evident in the organization of hair cells within the auditory and vestibular sensory epithelia where it is commonly called Planar Cell Polarity (PCP) (Tarchini and Lu, 2019). For each, the orientation of stereociliary bundles is coordinated between neighboring hair cells, and the population of cells are aligned relative to the functional anatomy of the sensory epithelium within which they reside. This two-fold organization of planar polarity occurs across diverse tissues and species. The third level of planar polarity is only found in the otolithic organs (utricle and saccule) of the vestibule (Deans, 2013) and the lateral line neuromasts of fish, where it was recently described as planar bipolarity (Kozak et al., 2020). In these systems, the hair cells are distributed between two groups that are aligned along a common polarity axis but differ in that their stereociliary bundles are oriented in opposite directions. For the utricle these groups are easily distinguished and meet at an intercellular boundary called the Line of Polarity Reversal (LPR). In the saccule and mature lateral line neuromasts the boundary constituting the LPR is less defined, and cells of opposite stereociliary bundle orientation may be intermixed. Previously planar bipolarity was proposed to be a manifestation of a broader tissue level or global organization (Deans, 2013). However, as mechanisms that establish planar bipolarity are tightly linked to intrinsic planar polarity, and do not appear to impact tissue-level PCP, this hierarchical organization seems less likely (Kindt et al., 2021). As I will describe, the mechanisms generating planar bipolarity would be better viewed as tissue-specific modulators of intrinsic cell polarity. These three aspects of inner ear planar polarity are mediated by separate signaling pathways encoded by different groups of polarity genes, but their coordinated activities are necessary for building functional auditory and vestibular sensory organs.

The goal of this review is to describe the commonalities and differences in hair cell planar polarity that occur in the auditory and vestibular structures of the vertebrate inner ear. In general, aspects of intrinsic planar polarity essential for mechanotransduction and planar cell polarity are shared between these modalities, while planar bipolarity has evolved to facilitate the specific functions of the otolithic organs. These areas of divergence make the inner ear a powerful system to study developmental mechanisms guiding planar polarity that can be applied to other systems. For example, the precise organization of stereociliary bundles in the organ of Corti allows for subtle mutant phenotypes to be rapidly identified, while planar bipolarity makes the otolithic organs an ideal system to study how overlapping signaling pathways modulate the output of planar polarization. This review will contain a combination of anatomical and physiological observations relating to the functional significance of planar polarity as well as molecular mechanisms that have been identified that build these systems, with differences and similarities between auditory and vestibular highlighted throughout.

## Functional Significance

### Auditory Hair Cells of the Mammalian Cochlea

**FIGURE 1:**
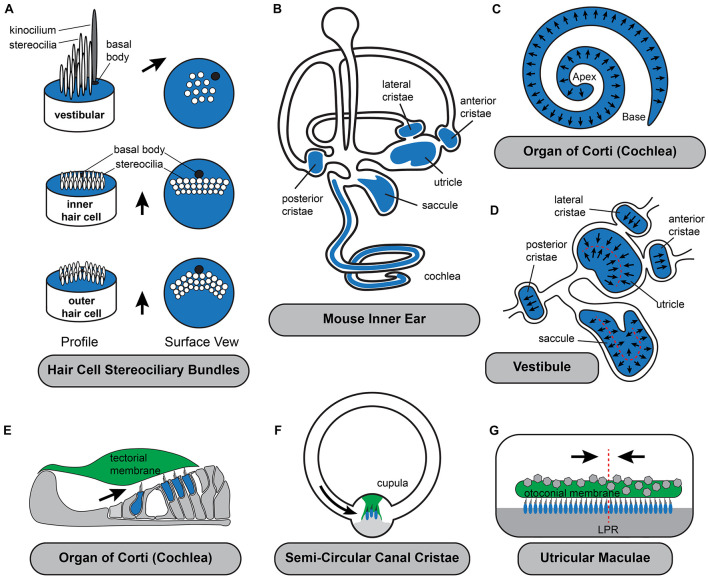
Planar polarization of the stereociliary bundle is coordinated with the anatomy of the inner ear. **(A)** The intrinsic polarization of auditory and vestibular hair cells is evident in the organization of stereocilia into rows of increasing height with the longest adjacent to the laterally displaced kinocilium and its associated basal body. While stereocilia length and organization varies the polarized organization is shared by all hair cell types. **(B)** Hair cells are located in the sensory epithelia (blue shading) with auditory hair cells located in the organ of Corti in the cochlea and vestibular hair cells distributed between the three semi-circular canal cristae and two otolithic organs, the utricle and saccule. **(C)** Throughout the organ of Corti, hair cells are oriented with their stereociliary bundles pointed toward the lateral edge of the cochlear spiral. **(D)** In the vestibule, the orientation of cristae hair cells is aligned with the associated semi-circular canal. In the utricle and saccule there is an array of stereociliary bundle orientations and the hair cells are divided between two groups patterned about the Line of Polarity Reversal (LPR, red dashed line). **(E–G)** Schematic cross-sections of the three types of sensory epithelia illustrating the direction of excitatory hair cell deflections. Stereociliary bundles are deflected by overlying extracellular matrices which are either fixed in place such as the tectorial membrane **(E)**, aligned with the associated semi-circular canals like the cupula of the cristae **(F)**, or in the **(G)** utricle and saccule are capable of moving in response to acceleration or tilt across 360 degrees of space. Black arrows in all panels illustrate the excitatory axis of stereociliary bundle deflection.

The proper orientation of hair cell stereociliary bundles relative to the anatomy of the cochlear duct is essential for their function as mechanosensory receptors (Figure 1). Auditory hair cells are located within the organ of Corti that spirals along the length of the mammalian cochlea. They are precisely patterned within four rows and surrounded by supporting cells that provide structural support. The most medial row contains Inner Hair Cells (IHC) that detect the sound contributing to auditory perception while the remaining three rows contain Outer Hair Cells (OHC) that facilitate frequency tuning and amplification mechanisms. The stereociliary bundles of both IHCs and OHCs are oriented with the excitatory axis of their bundles oriented parallel to the short axis of the cochlea and as a result, bundle deflections toward the lateral edge of the cochlear spiral are excitatory (Figure 1C). On IHCs the rows of stereocilia are flat with the kinocilium centrally positioned adjacent to the tallest row and laterally displaced on the cell surface. On OHCs the rows of stereocilia rows arranged in a V-shape with the kinocilium located at the vertex where it is also laterally displaced and adjacent to the tallest stereocilia. The kinocilium is a transient component of auditory bundles that is lost near the onset of hearing, likely to facilitate high frequency hearing (Elliott et al., 2018). However, the basal body that anchored the kinocilium is maintained in this lateral position for the life of the cell. OHC stereocilia contact the overlying tectorial membrane and are deflected when the overlying tectorial and underlying basilar membranes vibrate in response to sound. IHC stereocilia are similarly deflected by fluid flow beneath the tectorial membrane as the result of these motions (Schwander et al., 2010). Since the tectorial membrane is attached on its medial side to the spiral limbus and therefore fixed in position it is critical that the hair cell planar polarity axis is oriented parallel to the short axis of the cochlear spiral so that hair cells are properly stimulated by sound.

The relationship between the direction of bundle deflection and the physiological response of individual hair cells has been directly measured following mechanical stimulation *in vitro* (Hudspeth and Corey, 1977; Shotwell et al., 1981) and using calcium imaging in zebrafish (Kindt et al., 2021). In these studies, maximal responses were only found when the bundle was deflected toward taller stereocilia while deflections in the opposite direction were inhibitory due to the closure of channels that had remained open at rest. In general, hair cells do not depolarize when bundles are deflected orthogonal to the axis of the staircase because this motion fails to place adequate tension on the tip links. When modeled, the relationship between the vector of the mechanical stimulus and the hair cell response *in vitro* is best fit by a cosine relationship (Shotwell et al., 1981). As a result, auditory function is likely quite tolerant to minor changes in bundle orientation since deviations of 20 degrees in either direction are only predicted to produce a 6% reduction in hair cell activation (100%-Cos20). Unfortunately, it is not straightforward to test the impact of stereociliary bundle misorientation on auditory function in the hearing mouse because mutations producing these phenotypes are pleiotropic with phenotypes impacting supporting cell morphology (Copley et al., 2013) and hair cell innervation (Ghimire et al., 2018; Ghimire and Deans, 2019; Jean et al., 2019), in addition to neural tube closure (Curtin et al., 2003; Montcouquiol et al., 2003; Lu et al., 2004; Wang J. et al., 2006; Wang Y. et al., 2006) and kidney development (Derish et al., 2020; Torban and Sokol, 2021). Until the significance of bundle orientation can be tested in isolation, we are left with the scientific irony that the precise organization of auditory hair cell leaves the smallest of changes readily visible to the experimentalist, yet may only have minor impacts on auditory function.

### Vestibular Hair Cells of the Semi-Circular Canal Cristae

Similar to auditory hair cells, the vestibular hair cells in the semi-circular canal cristae must be coordinately oriented with the anatomy of their associated non-sensory structure, the semi-circular canal, for proper function. Cristae hair cells detect rotational accelerations of the head and mediate the vestibular ocular reflexes (VOR) essential for maintaining visual gaze (Fetter, 2007). These hair cells are distributed between the three cristae of the anterior, posterior and lateral semi-circular canals (Figures 1B,D,F). Cristae hair cells are stimulated by inertial fluid movements within the canals that occur in response to rotational head movements. These fluid movements impinge on an extracellular matrix called the cupula that surrounds and deflects the hair cell stereociliary bundles. As a result of this anatomical organization, the stereociliary bundles must be oriented with the excitation axis of their stereociliary bundles aligned parallel to the semi-circular canal in order for the hair cell to be stimulated by cupula movements. Therefore, the direction of motion that may be detected by cristae hair cells is determined solely by the orientation of the lateral, anterior or posterior canal. In addition, the hair cells are oriented in a single direction within the cristae so rotations in one direction are excitatory while rotations in the opposite are inhibitory. Since the two ears are mirror images of one another, rotational movements excite hair cells in the cristae of one ear while simultaneously inhibiting hair cells in the complementary cristae of the opposite ear. The full range of head rotations are perceived through the integration of cristae output from the left and right ears by the vestibular nucleus of the hindbrain. As a result, coordinating the planar cell polarity of hair cells within cristae and relative to the associated semi-circular canals is critical for vestibular function.

### Vestibular Hair Cells of the Utricle and Saccule

The sensory epithelia of the utricle and saccule contain hair cells that detect gravity and linear acceleration. Their stereociliary bundles contact an overlying extracellular matrix that is embedded with calcium carbonate crystals, called the otoconial membrane that moves in response to acceleration and has sufficient mass to detect gravity. Unlike the tectorial membrane of the cochlea or the cupula in the cristae, the otoconial membrane is not physically tethered to non-sensory structures and therefore is capable of deflecting vestibular hair cell in response to movements across a 360 degree range. The planar polarized organization of utricular and saccular hair cells reflects this because their stereociliary bundles are similarly distributed across a range of orientations (Figure 1D) while remaining tightly aligned with their nearest neighbors.

The most salient aspect of planar polarity in the otolithic organs is the division of hair cells between two groups aligned along a common PCP axis, but with oppositely oriented stereociliary bundles (Deans, 2013). In the utricle the bundles of the two groups are oriented with their kinocilia on the side of the cell pointed toward each other while in the saccule the bundles are oriented with their kinocilia pointed away. A similar planar bipolarity organization is seen in the lateral line of fish and has been studied extensive using the zebrafish model (Lopez-Schier and Hudspeth, 2006; Ji et al., 2018; Jacobo et al., 2019; Navajas Acedo et al., 2019; Kozak et al., 2020). As a result of this organization, stimuli will produce parallel streams of excitation and inhibition since movements of the tectorial membrane will deflect the bundles in opposite directions. These two groups are innervated by distinct populations of afferent neurons that transmit parallel streams of information through the VIIIth cranial nerve. In the mouse it has been further demonstrated that these streams have distinct central projections with neurons innervating the medial regions of the utricle projecting to the vestibular nuclei and neurons innervating the lateral region projecting to the cerebellum (Maklad et al., 2010). Afferent innervation in the zebrafish is also coordinated with bundle orientation because peripheral axons only contact hair cells of one type and maintain this preference even after the axon is severed and allowed to re-innervate the neuromast (Lozano-Ortega et al., 2018).

In the mouse utricle and saccule, the boundary between hair cells with oppositely oriented stereociliary bundles is described as the line of polarity reversal (LPR). There is not a distinct landmark or structure that constitutes the LPR, and often two hair cells with opposing bundle orientations are separated by just a single intervening supporting cell. The LPR is closely associated with the striola, a physiologically specialized area in the medial region of the maculae enriched with hair cells contacted by afferent neurons that form complex calyceal endings surrounding multiple hair cells. The striolar region can be molecularly distinguished because striolar hair cells express the calcium binding protein oncomodulin while the afferent neurons innervating them express calretinin. In the mouse utricle the LPR runs along the lateral edge of the striolar region and occasionally these features are described synonymously despite being anatomically and developmentally distinct. For example, the LPR actually passes through the striolar region of the mouse saccule and during development the striolar region is positioned by a gradient of retinoic acid signaling that is independent of the LPR which persists when this gradient is disrupted (Desai et al., 2005; Ono et al., 2020). The LPR is also variable in its organization and can appear as a smooth boundary when mapped in the mouse utricle and a jagged boundary in the mouse saccule where hair cells with oppositely oriented bundles are more often interspersed at this border (Stoller et al., 2018).

In addition to planar bipolarity, the organization of stereociliary bundles in the maculae differs from the cochlea and the cristae because bundle orientation gradually radiates across the width of the sensory epithelia. As a result, even for hair cells restricted to one side of the LPR, the orientation of individual bundles located on opposite sides of the sensory epithelia may differ by more than 100 degrees (Figure 1D). Since the range of motion that can be detected by an individual hair cell is determined by the orientation of its stereociliary bundle, the planar polarity organization of the vestibular maculae results in individual cells that are orientated to be optimally stimulated by motions spanning a 360 degree range. Furthermore, the overlying otoconial membrane is not anchored within the vestibule, therefore linear accelerations and tilt can provide stimuli throughout this function range. Thus, unlike the cristae where the direction of rotational acceleration that can be detected is determined by the position of the semi-circular canals, in the vestibular maculae the range of linear acceleration that can be detected is determined by the organization of the mechanosensory hair cells themselves. Theoretically this organization could constitute a sensory representation of motion across 360 degrees of space, although the level of resolution maintained by the utricle or saccule have not been established, and likely differs between species with different locomotive behaviors. Foremost, the resolution imparted by an individual hair cell is diminished by the cosine rule and mechanical characteristics of bundle deflection (Shotwell et al., 1981). It is also impacted by the morphology of afferent neurons which branch and contact multiple neighboring hair cells which may have a modest range of bundle orientations depending on their distribution (Eatock and Songer, 2011). Despite these anatomical considerations, it remains clear that the organization of bundle orientations allow the vestibular maculae to resolve motions in opposite directions and that this resolution is maintained in their central projections to either the vestibular nuclei or the cerebellum. Regardless of the level of resolution, the planar polarized organization of hair cell stereociliary bundles in the maculae establishes a sensory representation of linear acceleration and gravitational tilt within the peripheral end organ and this representation is dependent upon planar polarity.

## Developmental Mechanisms

The developmental mechanisms that guide hair cell planar polarity are generally shared between auditory and vestibular hair cells. The differences that do occur underlie specialized evolutions unique to the structure and function of the sensory epithelia in which the hair cells reside rather than functional differences between the hair cells themselves. As a result, greater differences in planar polarity occur between vestibular hair cells of the maculae and the semi-circular canal cristae than between the hair cells of the cristae and cochlea. Mechanisms guiding hair cell planar polarity development can be broadly divided between three signaling pathways acting at distinct anatomical scales (Deans, 2013). Common to all hair cells is the cell intrinsic polarization that directly regulates morphogenesis of the stereociliary bundle. The second is planar cell polarity, a tissue level organization that aligns cell intrinsic polarities along a common polarity axis. The third level of organization is unique to the otolithic organs and distinguishes hair cells in the utricle and saccule from the other sensory organs of the inner ear. This organization is epithelial planar bipolarity, and describes the division of hair cells between two groups patterned about the LPR. Planar bipolarity is also seen in the organization of neuromast hair cells of the zebrafish lateral line and developmental events guiding this process are conserved between systems (Jiang et al., 2017; Kozak et al., 2020; Kindt et al., 2021). Additional features distinguishing auditory and vestibular hair cells that are not directly related to bundle polarity, including stereociliary length and retention of the kinocilium will not be discussed in detail.

### Cell Intrinsic Signaling Pathways Polarize the Stereociliary Bundle

Within an individual hair cell, planar polarity is evident in the organization of the stereociliary bundle, and the position of the kinocilium with its associated basal body on the apical cell surface (Figure 1A). Consistent with this, the signaling mechanisms guiding intrinsic planar polarity are closely linked to actin-based mechanisms regulating stereocilia length and staircase formation, and the cytoskeletal regulators that position the basal body at the lateral edge of the apical cell surface (Grimsley-Myers et al., 2009; Sipe and Lu, 2011; Tarchini et al., 2016; Tadenev et al., 2019). Thus, mechanisms guiding cell intrinsic polarity regulate both the actin and tubulin cytoskeletal networks. Morphological observations made during hair cell development and differentiation suggest a sequential model in which cell intrinsic polarization is guided by the kinocilium with crosslinks between the kinocilium and the adjacent stereocilia regulating the actin-based processes determining stereocilia length (Tilney et al., 1992; Lefevre et al., 2008; Richardson and Petit, 2019). Consistent with this idea, the basal body appears in a lateral position before stereocilia elongation and staircase formation (Denman-Johnson and Forge, 1999). Experimentally it is also common to rely exclusively on the position of the basal body for defining hair cell polarity because it occurs early and is not impacted by disruptions of bundle morphology. This application has only reinforced acceptance of the sequential model of bundle orientation. However, more recent genetic studies (discussed below) suggest that basal body positioning and asymmetric actin dynamics are established independently and that subsequent interactions between the kinocilium and stereocilia actually reinforce and maintain the polarized structure of the bundle. Consistent with these observations, when intrinsic polarity is disrupted the kinocilium is often dissociated from the stereocilia which themselves may assemble in malformed or disorganized rows (Tarchini and Lu, 2019).

One distinctly polarized signaling pathway is the Par3-Rac1-PAK cascade which was one of the first associated with the development of intrinsic polarity (Grimsley-Myers et al., 2009). Intrinsic polarity is significantly disrupted following mutations in the small GTPase Rac1, with kinocilia that are dissociated from the stereocilia and malformed bundles that are frequently fragmented. Although Rac1 is present throughout auditory hair cells, its downstream effector PAK (P21 activated kinase) is only detected in its phosphorylated active state along the lateral cell boundary. PAK is a known regulator of cytoskeletal dynamics (Bokoch, 2003) and in this lateral location activated PAK is in the vicinity of the sub-apical microtubule network and kinocilium, supporting a model in which interactions between the basal body and hair cell junction are stabilized by cortical Rac-PAK signaling (Grimsley-Myers et al., 2009). Interestingly, the lateral activation of PAK by Rac1 is dependent upon Par3, a polarity molecule better known for its function opposite of the Par6/aPKC complex during apical-basolateral polarization of epithelial cells. This pathway is also active in hair cells where it patterns the apical cell surface, and Par3 is required for Rac1 activation of PAK. Conversely, expression of an activated Rac1 rescues the *Par3* mutant phenotype demonstrating that these molecules act in a linear path to regulate intrinsic polarity (Landin Malt et al., 2019).

**FIGURE 2:**
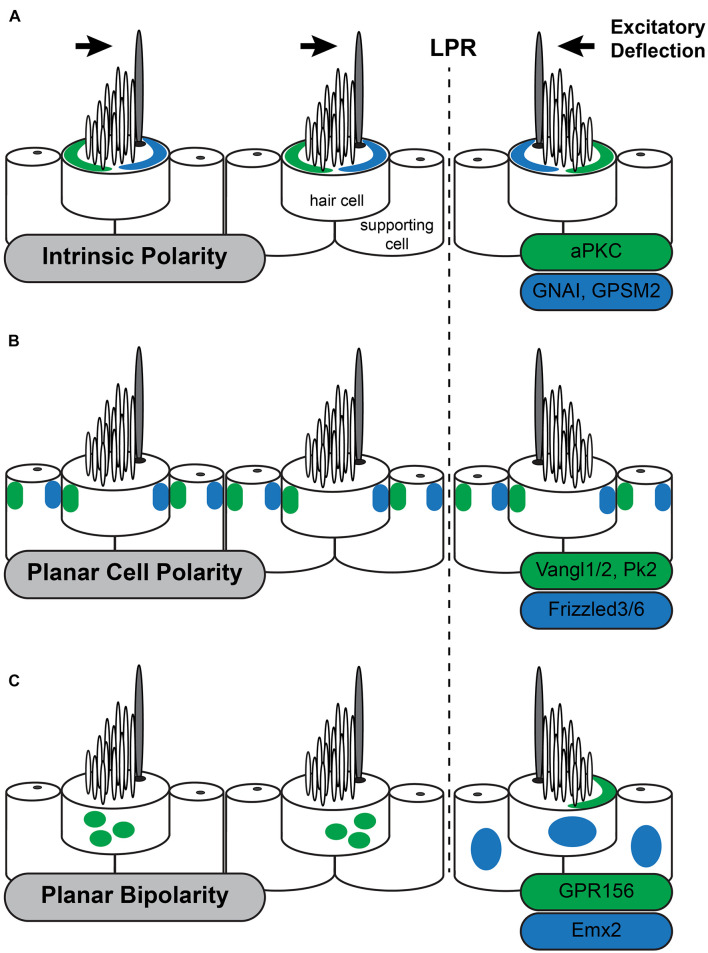
Three planar polarity signaling pathways organize stereociliary bundles in the utricle. The three levels of planar polarity organization in the sensory epithelia of the mouse inner ear utilize distinct sets of signaling proteins during their development. Each set of proteins is uniquely distributed throughout the sensory epithelia or within hair cells. **(A)** The Intrinsic polarity factors GNAI/GPSM2 are enriched at the apical cell surface in the stereocilia bare zone adjacent to the kinocilium while aPKC is enriched on the opposite side of the cell surface. The relative position of GNAI/GPSM2 and the kinocilium is constant and as a result the position of GNAI/GPMS2 is reversed in hair cells on opposite sides of the Line of Polarity Reversal (LPR) similar to stereociliary bundle orientation. **(B)** The Planar Cell Polarity proteins Vangl1/2 and Pk2 are asymmetrically distributed opposite of Fzd3/6 within hair cells and supporting cells throughout the sensory epithelia of the utricle and saccule. This distribution is unchanged at the LPR, and as a result the kinocilium is adjacent to Fzd3/6 in the medial utricle and opposite of Fzd3/6 in the lateral utricle. **(C)** Planar Bipolarity and the position of the LPR are established by the transcription factor Emx2 which is expressed in hair cells and supporting cells throughout the lateral utricle. GPR156 is expressed in all hair cells but is only present delivered to the apical cells surface in hair cells that also express Emx2. In these cells GRP156 is always enriched at the cell boundary opposite of the kinocilium.

The most distinctive feature of intrinsic polarity is the staircase array of stereocilia suggesting that factors regulating filamentous actin assembly should have an important role in regulating intrinsic hair cell polarity. Therefore, it is surprising that a set of molecules typically associated with mitotic spindles and the regulation of cortical force during cell division contributes to this aspect of intrinsic planar polarity in hair cells (Morin and Bellaiche, 2011). This includes the G-protein inhibitory subunit GNAI (Tarchini et al., 2013) and its associated regulatory protein GPSM2 (also known as LGN) (Ezan et al., 2013). GPSM2 maintains GNAI in a GDP-bound state and this complex is enriched near the lateral hair cell boundary early in development (Figure 2A). Although this localization coincides with the lateral movement of the basal body these processes are not spatially coordinated making it unlikely that either one guides the other. GNAI/GPSM2 sculpts formation of the stereociliary bundle by establishing a microvilli free zone on the apical cell surface between the lateral cell boundary and the kinocilium, promoting extension of the longest row of stereocilia, and redistributing Par3 and aPKC to lateral and medial positions, respectively (Tarchini et al., 2013; Tadenev et al., 2019). Patterning of the apical cell surface by Par3 and aPKC promotes formation of a compact stereociliary bundle at the region between. This apical blueprint is maintained through neonatal development in the mouse by the multiple PDZ domain (MPDZ) protein which is required to maintain segregation of GNAI/GPSM2 and aPKC (Jarysta and Tarchini, 2021). Despite these advances it remains unclear what initiates the polarized distribution of GNAI-GPSM2 within hair cells nor whether GNAI-GPSM2 regulates Par3 upstream of the Par3-Rac1-PAK cascade.

Subsequent to polarization, maintenance of the kinocilium at the lateral edge of the apical cell surface is dependent upon a microtubule array assembled beneath the cell surface. The distribution of the microtubule binding protein EB1 in auditory hair cells indicates that the microtubule plus-ends are enriched at the lateral edge of auditory hair cells where the basal body is located (Ezan et al., 2013). Moreover, disruptions of the microtubule network following mutation of the cytosolic dynein regulatory gene *Lis1* results in displaced basal bodies and associated stereociliary bundle defects (Sipe et al., 2013). The kinesin motor component Kif3a, better known for its role in intraflagellar transport, also contributes to protein trafficking along microtubule networks in hair cells and contributes to the lateral Rac1-PAK signaling pathway (Sipe and Lu, 2011). In its absence auditory hair cells also have displaced basal bodies and stereociliary bundle defects. However, neither Lis1 or Kif3a mutations impact the initial movement of the basal body to the lateral edge suggesting instead that the microtubule network is necessary for maintaining rather than initiating intrinsic polarity. As the basal body is a well known microtubule organizing center (MTOC) it would be logical that once asymmetrically positioned it would maintain this localization through the microtubule cytoskeleton. Lastly, while the kinocilium and stereocilia appear to be positioned independently, subsequent interactions between these structures likely reinforce their polarization and maintain cohesion of the bundle (Lefevre et al., 2008).

### Planar Cell Polarity Signaling Establishes and Maintains Tissue Polarity

Hair cell planar cell polarity is established by intercellular signals that coordinate the orientation of neighboring cells and a global organizer that orients the population within the sensory epithelia. The most salient example of inner ear planar polarity is the coordinated orientation of stereociliary bundles between neighboring hair cells in the organ of Corti. The genes regulating this coordination were first identified in *Drosophila* and described the organization of epidermal cells on the wing and body that project trichomes or hairs toward the wing tip and tail (Vladar et al., 2009; Goodrich and Strutt, 2011). *Drosophila* PCP genes encode the Wnt receptor Frizzled, the transmembrane protein Van Gogh and the seven-pass transmembrane cadherin Flamingo/Starry Night which are asymmetricaly distributed at apical cell boundaries with Frizzled and Flamingo or Van Gogh and Flamingo enriched at opposite poles. The cytoplasmic proteins Disheveled and Diego colocalize with Frizzled, while Prickle colocalizes with Van Gogh. In general, it is thought that transmembrane proteins form an intercellular signaling complex that coordinates polarity between neighboring cells while cytoplasmic factors contribute to intracellular feedback that reinforces the molecular polarization of individual cells.

The initial suggestion that PCP genes could function in the inner ear was presented in an essay by Julian Lewis and Alex Davies (Lewis and Davies, 2002). They predicted that, based on their anatomical organization, hair cell polarization would require multiple levels of organization and that PCP signaling was appropriate for coordinating the orientation of intrinsic polarities between neighboring hair cells. The conservation of PCP gene function between *Drosophila* wing hairs and inner ear hair cells was subsequently demonstrated by Curtin et al. (2003) and Montcouquiol et al. (2003) who observed that *Vangl2*, the vertebrate ortholog of the *Drosophila* gene *vang gogh*, and *CELSR1*, the vertebrate ortholog of *flamingo/starry night* were required for the coordinated alignment of auditory hair cell stereocilia. Although they impacted different populations of hair cells, a common attribute of these phenotypes was that the intrinsic polarization of the stereociliary bundle was maintained while stereociliary bundles were misoriented between neighboring cells and the tissue axis. This is in contrast with *Drosophila* phenotypes where both intrinsic and tissue polarity are disrupted, with the cell’s hair or basal body appearing in a central location on the apical cell surface (Carvajal-Gonzalez et al., 2016; Garrido-Jimenez et al., 2018). This difference is likely due to the intrinsic polarity pathways that are unique to hair cells (Tarchini and Lu, 2019). PCP signaling in the vertebrate ear coordinates intrinsic polarity between neighboring cells and this signal is relayed by an intercellular signaling complex comprised of Vangl1 and Vangl2 (Vangl1/2) (Montcouquiol et al., 2003; Yin et al., 2012; Stoller et al., 2018), Frizzled3 and Frizzled6 (Fzd3/6) (Wang Y. et al., 2006) receptors with their associated transduction partner Disheveled1/2/3 (Wang J. et al., 2006), and the atypical cadherin CELSR1/2/3 (Flamingo in *Drosophila*) (Curtin et al., 2003; Duncan et al., 2017; Figure 2B). These factors are conserved between vertebrates and invertebrates and have been described as the Core PCP proteins. Common phenotypes to core PCP mutants are hair cell polarity defects and profound neural tube defects called craniorachischisis in which the neural tube fails to close along the entire length of the body. Craniorachischisis is also seen in *Scribble* (Scrb) (Murdoch et al., 2001) and *Ptk7* mutants (Lu et al., 2004) which indicates a role in PCP signaling though these functions appear to be vertebrate specific. In contrast the vertebrate ortholog of Drosophila Prickle (Prickle-like2, PK2) is asymmetrically localized in hair cells and supporting cells of the inner ear like other PCP proteins (Deans et al., 2007) but bundle orientation phenotypes have not been reported for *Pk2* mutants nor identified in *Pk1* mutant mice (Yang et al., 2017). One explanation may be that Prickle functions in *Drosophila* to reinforce intracellular polarization but that is redundant with the intrinsic polarization mechanisms functioning in hair cells.

Several lines of evidence argue that that PCP signaling functions in parallel to the intrinsic planar polarity and planar bipolarity signaling pathways in hair cells. Foremost, the asymmetric distribution of PCP proteins is not changed when intrinsic signaling through the GNAI pathways is disrupted (Tarchini et al., 2013). Similarly, in *Rac1* conditional knockouts Fzd3 is asymmetrically distributed at cellular junctions during early development (prior to E17.5) though it is lost during subsequent hair cell maturation (Grimsley-Myers et al., 2009). The changes in Frizzled localization in *Rac1* cKOs are not sufficient to produce phenotypes resembling *Fzd3/6* mutants (Wang Y. et al., 2006). However, these changes do suggest there may be some cross talk between the pathways. Conversely, in PCP mutants hair cells are misoriented relative to each other yet continue to form polarized stereociliary bundles, and the intrinsic factors remain polarized and are properly distributed relative to those bundles (Grimsley-Myers et al., 2009; Tarchini et al., 2013). PCP signaling also appears independent of planar bipolarity because the asymmetric distributions of PCP proteins do not change at the LPR or in mutants with disrupted planar bipolarity (Deans et al., 2007; Jiang et al., 2017). This has led to the general view that PCP signaling functions as a compass that coordinates the orientation of individual cells relative to the tissue axes rather than an effector directing polarization of the stereociliary bundle.

The evidence that core PCP proteins mediate intercellular signaling is based on the domineering non-autonomy phenotypes seen in *Drosophila* where the orientation of wild type cells is disrupted if they are adjacent to PCP mutant clones. When similar mutant boundaries are generated in the vestibular maculae using restricted expression of Cre recombinase, the adjacent wild type cells are also misoriented and turn away from the boundary (Stoller et al., 2018). Similar domineering non-autonomy phenotypes have been seen after Vangl2 overexpression in the chick basilar papillae (Sienknecht et al., 2011) and transplant experiments in the frog epidermis that evaluated the planar polarity of multiciliated cells (Mitchell et al., 2009). In each of these systems wild type cells became misoriented when neighboring experimental cells in which PCP signaling is manipulated, demonstrating the conservation of intercellular signal between vertebrates and *Drosophila*. Signaling is dependent upon the asymmetric distribution of Frizzled and Vangl2 to opposite sides of cells and polarity signals are relayed across intercellular space by Frizzled and Vangl2 acting as a receptor ligand pair (Wu and Mlodzik, 2008) or *via* Flamingo/CELSR bridges (Struhl et al., 2012). Surprisingly, this complex appears to be restricted to just one side of cochlear hair cells with Frizzled3 on the medial side of the hair cell and Vangl2 on the lateral side of the adjacent supporting cell (Wang Y. et al., 2006; Giese et al., 2012). In contrast, immunofluorescent labeling suggests that PCP proteins are on both sides of supporting cell junctions (Duncan et al., 2017). One interpretation of these distributions is that PCP is communicated across inner ear sensory epithelia by supporting cells, and that hair cells receive polarization cues without contributing to their propagation. In the vestibular system it is unclear whether PCP guides supporting cell development because they lack distinct structural features whereas in the organ of Corti where supporting cells have more structural complexity it is clear that Vangl2 is required for their polarized development (Copley et al., 2013).

The outstanding unanswered question for both the auditory and vestibular sensory epithelia is what determines the tissue-wide organization and acts as the global organizer of PCP. For example, in the cristae what aligns the PCP axis with the semi-circular canal, and how does this signaling mechanism establish the opposite orientation of hair cells in the lateral and anterior cristae (Figure 1D). Intercellular signaling suggests that once initiated, PCP could be relayed to new cells or maintained during cellular movements and that an organizer could be transient. It had been anticipated that an extracellular morphogen would be this cue, with Wnts proposed as a ligand since the Wnt receptor Frizzled is also a core PCP protein (Yang and Mlodzik, 2015). However, when Wnt is removed from the inner ear, planar polarity is only mildly impacted (Ghimire and Deans, 2019; Najarro et al., 2020). Similarly, in *Drosophila* when all *Wnt* genes are deleted there is no impact on epithelial wing cell PCP (Ewen-Campen et al., 2020; Yu et al., 2020). While Wnts may not define the tissue PCP vector for the inner ear, recently findings suggest that non-canonical Wnt/G-protein signaling may instead act to coordinate cell-intrinsic and tissue-level PCP (Landin Malt et al., 2020, 2021). Thus, instead of establishing bundle orientation, Wnts may function to refine bundle orientation, particularly for the last row of OHCs (Dabdoub, 2003). Because PCP is reinforced and propagated by intercellular signaling an exciting hypothesis is that signals polarizing the early otocyst establish a PCP axis in the poresensory domain that is maintained throughout morphogenesis (Deans, 2013).

### Planar Bipolarity Distinguishes the Vestibular Maculae and Lateral Line

Vestibular hair cells in the utricle and saccule are distributed between two groups with oppositely oriented stereociliary bundles that meet at the LPR. In the utricle, hair cells are organized so that their stereociliary bundles are pointed toward each other while in the saccule the bundles point away, but in either case, the hair cells are oriented along the same PCP axis. Similar organization is seen in the lateral line neuromast of zebrafish where hair cells are oriented along a common axis yet are divided into two groups with stereociliary bundles oriented in opposite directions. While previously described as a readout of global planar polarity (Deans, 2013), planar bipolarity is more likely a specialized from of intrinsic polarity that is dependent upon the PCP axis that underlies tissue polarity. Moreover, as described in greater detail below, the LPR is now known to be established through the regulation of cell intrinsic events rather than global patterning mechanisms.

The LPR is not organized by PCP signaling because the subcellular distribution of these factors is not altered at the LPR (Deans et al., 2007). Instead, the LPR is formed by transcriptional activity of Emx2 which is expressed by hair cells and supporting cells in the lateral region of the utricle (Figure 2C) and medial region of the saccule in a pattern matching the LPR. In the absence of Emx2 hair cells point in a single direction while overexpression using viral vectors or genetic tools in mouse gives the opposite result (Jiang et al., 2017). Since the distribution of PCP proteins are not altered in *Emx2* mutants, this transcription factor is likely to act as a molecular switch regulating intrinsic polarity. However, Emx2 is also expressed in the cochlea and mutant phenotyping reveals developmental roles extending beyond planar polarity because the absence of Emx2 OHCs fail to differentiate (Holley et al., 2010). The basis of this phenotype has not been determined though one possibility is that Emx2 influences regional patterning and that auditory and vestibular hair cell phenotypes emerge because cells in these regions are not properly differentiated. Consistent with this possibility, the vestibular macuale are smaller in one Emx2 mutant line (Holley et al., 2010), though this has not been apparent in others where macular area was measured (Jiang et al., 2017). Thus, it remains unclear whether Emx2 acts atop a gene regulatory network that establishes regional identity with bundle polarity being one downstream feature, or if Emx2 directly regulates the expression of effectors that determine stereociliary bundle orientation.

An important downstream effector of Emx2 function is the orphan G-protein coupled receptor GPR156 which gives similar phenotypes to Emx2 when mutated (Kindt et al., 2021). Interestingly GPR156 cannot be a direct Emx2 target because GPR156 is expressed in all hair cells. Instead it is only functional in cells expressing Emx2 where it is delivered to the apical surface suggesting that GPR156 trafficking pathways are regulated by Emx2. In cells on the opposite side of the LPR, GPR156 is not apical and appears to be retained in intracellular organelles (Figure 2C). Remarkably pertussis toxin-mediated inhibition of GNAI suggest that these G-proteins act downstream of GPR156 because blocking GNAI function in this way gives phenotypes resembling both *Emx2* and *GPR156* mutants. Further genetic interaction studies confirm a functional hierarchy of Emx2, GPR156 and GNAI. This study further suggests that GNAI has parallel functions in regulating intrinsic polarity and planar bipolarity because the regulatory protein GPSM2 is not required for planar bipolarity. An exciting possibility is that GNAI could function in an activated GTP-bound state downstream of GRP156 though this has not been demonstrated. Regardless, the function of this Emx2-GPR156-GNAI signaling cascade is to determine the position of the kinocilium relative to the PCP axis. In cells expressing Emx2 the kinocilium is always opposite of Fzd3/6 as seen in the organ of Corti (Wang Y. et al., 2006) and inferred from the distribution of PCP proteins in supporting cells of the utricle (Deans et al., 2007). Thus, Emx2-dependent planar bipolarity regulates how intrinsic planar polarity is organized relative to the planar cell polarity axis.

## Cellular Movements

One aspect of inner ear development that distinguishes auditory from vestibular hair cells are the dynamic cellular movements that pattern the precisely aligned rows of hair cells within the organ of Corti. Evidence from live imaging suggests that cochlear extension occurs through radial intercalation, directional migration of supporting cells and tissue growth (Driver et al., 2017). The precisely aligned rows of hair cells are generated through convergent extension movements in which newly specified hair cells converge along the radial axis and extend along the long axis of the cochlear spiral. Convergent extension is the result of collective cell migrations and are seen in multiple morphogenic events during embryonic development that include gastrulation and lengthening of the body axis prior to neuralation. During neural tube closure, cellular convergence at the midline lengthens the anterior:posterior body axis and brings the neural folds into closer proximity so that they may fuse to form the dorsal most neural tube. The directed cell migration underlying convergent extension movements requires PCP signaling, and when that is disrupted polarized cellular movements are randomized. As a result, a common phenotype associated with vertebrate PCP gene mutations including *Vangl2*, *Dsh*, and *Fzd3/6* is an open neural tube defect extending from the midbrain-hindbrain boundary to the tail called craniorachischisis (Copp et al., 2003; Greene et al., 2009).

Similar disruptions in the convergent extension movements of hair cells in *vangl2*, *Dsh* and *Fzd3/6* double mutants have been attributed to the formation of supernumerary rows of OHCs in the cochlear apex, and cochlea that are significantly reduced in length (Montcouquiol et al., 2003; Wang J. et al., 2005; Wang Y. et al., 2006). One interpretation of these phenotypes is that PCP dependent convergent extension of hair cells drives cochlear duct extension. Indeed, organ of Corti cells appear to migrate away from the apical end of cochlear ducts when grown *in vitro* (Wang J. et al., 2005). However, these mutant phenotypes are pleiotropic and it is also possible that cochlear phenotypes are secondary to craniorachischisis which disrupts morphogenesis of the inner ear because they are displaced in a medial-lateral orientation within the embryo, and because they are exposed to embryonic fluids of the yolk sac. Indeed, for *vangl2* CKOs in which craniorachischisis does not occur, the length of the cochlear duct is not altered and supernumerary OHC rows cannot be detected despite comparable changes to stereociliary bundle orientation to *Looptail* mutants (Copley et al., 2013). This *vangl2* CKO phenotype is not consistent with hair cell convergence producing a driving force that extends the cochlear duct, and a simpler explanation is that PCP signaling keeps cells properly oriented relative to each other as they undergo large collective movements. A definitive experiment to distinguish these alternatives would be to analyze hair cell patterning in a CKO in which gene deletion is restricted to the dorsal neural tube thereby producing craniorachischisis without impacting PCP gene expression in the ear itself.

The relative orientation of the stereociliary bundle is also dynamic and auditory stereociliary bundle orientation can change over time. While it is not clear whether this involves rotation of apical cell components or rotational movements of the entire hair cell, rotational movements have been inferred in both wild type and mutant states. This is most obvious for *vangl2* CKO where misoriented bundles are largely corrected during early postnatal development (Copley et al., 2013). However, in wild type mice the bundles atop third row OHCs also reorient over this time through a refinement process that involves Wnt (Dabdoub, 2003). The vertebrate PCP factor PTK7 may also play an important role in reorienting cells so that they are aligned along a common planar polarity axis by regulating actomyosin contractility at hair cell and supporting cell junctions (Lee et al., 2012; Andreeva et al., 2014). Despite these mechanisms, not all hair cells are capable of rotation. For example hair cells in the cochlear apex are never corrected in *vangl2* CKOs (Copley et al., 2013) and first row OHCs never reorient in *GPR156* mutants (Kindt et al., 2021). An interesting explanation is that shear forces that organize and pattern supporting cells in the abneural organ of Corti also drive hair cell rotation which is why it is a phenomenon primarily seen for the third row of OHCs (Cohen et al., 2020).

Hair cells develop in a gradient from base to apex with progressive maturation of bundle characteristics, gene expression and innervation. Similarly, the final orientation of hair cells is progressive, particularly for OHCs with bundles rotating from an initial tilt toward the cochlear apex toward the abneural boundary. This has significant experimental consequences if not properly controlled. For example, since bundles tilt toward cochlear apex, and cochlea chirality differs between left and right ear, samples from opposite ears cannot be pooled for quantification unless images from one ear are “mirrored” prior to analysis. Furthermore, because the cochlea develops as a gradient from base to apex, bundle orientation can differ significantly between OHCs located in the base versus the apex, and between cochlea harvested at different developmental stages. As a result, mutant models where overall embryo development is delayed often show OHC bundle orientations that accurately reflect their developmental stage but may be mistaken for a planar polarity phenotype when compared to fully developed littermate controls.

## Outstanding Questions

Comparing planar polarity development and function between the auditory and vestibular systems has led to significant advances in our understanding of the molecular mechanisms guiding cellular polarization at the intrinsic and tissue levels. Despite this progress there remain several outstanding questions. Foremost, is there a global organizer that establishes the initial PCP axis, and does this organizer have a conserved role across different species and planar polarity model systems? Current work from *Drosophila* indicates that Wnt is not likely to have a role in that process and an exciting possibility is that alternatives could be identified by research in the inner ear. For example, morphogens that contribute to patterning the major axes of the developing otocyst that include BMP, Sonic Hedgehog and Retinoic Acid. One possibility is that a polarity axis is established by one of these morphogens in the early prosensory domain that is maintained throughout morphogenesis by interecellular PCP signaling.

It will also be important to establish whether or not regenerated hair cells, whether they are stem cell derived or endogenous, are capable of establishing these three levels of planar polarity. Will hair cells re-introduced into damaged tissue be able to adopt a proper orientation for their organ and location? And will this support auditory and vestibular function? A compelling set of regeneration experiments in the chick suggest this may the case and that PCP proteins in supporting cells are available to guide the planar polarization of new hair cells (Warchol and Montcouquiol, 2010). For the vestibular system, how is bundle orientation correlated with innervation and will reinnervation respect LPR? Studies in zebrafish suggest that bundle orientation and hair cell identity are linked to innervation patterns and that reinnervation mechanisms will be resilient and accurate (Pujol-Marti et al., 2014) though whether this is maintained in mammals is yet to be determined. While it is clear there remains significant work to be done to answer these questions, the breadth of studies completed to date demonstrate that we remain pointed in the right direction.

## Author Contributions

MRD wrote, revised, and reviewed the manuscript.

## Conflict of Interest

The author declares that the research was conducted in the absence of any commercial or financial relationships that could be construed as a potential conflict of interest.

## Publisher’s Note

All claims expressed in this article are solely those of the authors and do not necessarily represent those of their affiliated organizations, or those of the publisher, the editors and the reviewers. Any product that may be evaluated in this article, or claim that may be made by its manufacturer, is not guaranteed or endorsed by the publisher.
